# Applications of Near Infrared Spectroscopy and Mirror Therapy for Upper Limb Rehabilitation in Post-Stroke Patients: A Brain Plasticity Pilot Study

**DOI:** 10.3390/jcm13216612

**Published:** 2024-11-04

**Authors:** Caterina Formica, Simona De Salvo, Nunzio Muscarà, Lilla Bonanno, Francesca Antonia Arcadi, Viviana Lo Buono, Giuseppe Acri, Angelo Quartarone, Silvia Marino

**Affiliations:** 1 IRCCS Centro Neurolesi Bonino Pulejo, 98124 Messina, Italy; katia.formica@irccsme.it (C.F.); nunzio.muscara@irccsme.it (N.M.); lilla.bonanno@irccsme.it (L.B.); francesca.arcadi@irccsme.it (F.A.A.); viviana.lobuono@irccsme.it (V.L.B.); angelo.quartarone@irccsme.it (A.Q.); silvia.marino@irccsme.it (S.M.); 2 Dipartimento di Scienze Biomediche, Odontoiatriche, e delle Immagini Morfologiche e Funzionali, Università degli Studi di Messina, c/o A.O.U. Policlinico ‘G. Martino’ Via Consolare Valeria 1, 98125 Messina, Italy; giuseppe.acri@unime.it

**Keywords:** spectroscopy, rehabilitation, stroke, neurological disorder, mirror therapy

## Abstract

**Objectives:** The aim of this study was to identify the neural pattern activation during mirror therapy (MT) and explore any cortical reorganization and reducing asymmetry of hemispheric activity for upper limb rehabilitation in post-stroke patients. **Methods:** A box containing a mirror was placed between the arms of the patients to create the illusion of normal motion in the affected limb by reflecting the image of the unaffected limb in motion. We measured the cerebral hemodynamic response using near-infrared spectroscopy (NIRS). We enrolled ten right-handed stroke patients. They observed healthy hand movements in the mirror (MT condition) while performing various tasks (MT condition), and then repeated the same tasks with the mirror covered (N-MT condition). **Results:** Significant activation of some brain areas was observed in the right and left hemiparesis groups for the MT condition, while lower levels of activation were observed for the N-MT condition. The results showed significant differences in hemodynamic response based on oxygenated (HbO) concentrations between MT and N-MT conditions across all tasks in sensorimotor areas. These neural circuits were activated despite the motor areas being affected by the brain injury, indicating that the reflection of movement in the mirror helped to activate them. **Conclusions:** These results suggest that MT promotes cortical activations of sensory motor areas in affected and non-affected brain sides in subacute post-stroke patients, and it encourages the use of these tools in clinical practice.

## 1. Introduction

Stroke is the most common cause of death and a leading cause of disability worldwide [1,2,3]. The prevalence of stroke in people aged 65–84 years is 6.5% (7.4% in males and 5.9% in females) [4,5,6]. Worldwide, it is estimated that 1/6 of people will experience a stroke in their lifestyle [7,8]. Stroke can result in a broad range of functional deficits, from upper and lower limb paresis to serious neurological and cognitive problems [9,10,11]. Various types of rehabilitative interventions are available to recover from motor deficits, such as robotic, bilateral extremity training and task-oriented approaches, among others [12,13]. Rehabilitation strategies are required to be repetitive, intensive, and task-specific for neuroplasticity to induce recovery [14]. In recent years, therapeutic approaches that required some degree of voluntary movement were used as well as the mirror therapy (MT) approach that was used in stroke patients with motor impairment. MT uses visual rather than somatosensory feedback to produce a desired response in the affected limb [15]. When the patients look in a mirror, the movement of the non-affected arm masks the view of the affected upper limb, eliciting the phenomenon of mirror illusion. In recent years, several studies have demonstrated the beneficial effects of MT on upper limb motor functions and activity of daily living [16], pain for patients with stroke [17,18], and for reducing phantom limb pain. An MT study with Virtual Reality (VR) support showed that MT combined with VR has potential effects on restoring the upper extremity motor function for chronic stroke patients, reinforcing multisensory integration with the effect of visual feedback and bilateral proprioceptive signals [19]. The outcome was probably determined by the neural mechanisms mediating the effect of MT. Some studies assumed that the Mirror Neuron System (MNS) plays an important role in the effect of MT [20,21]. Studies conducted on healthy subjects observed differences in the cerebral activation patterns during movement observation and movement mirroring, demonstrating activation in the contralateral Precuneus (PC) to the patient’s perceived own hand, while the observation of hands of another person showed no such lateralization [21]. The same results were found in stroke patients with a severe paresis of the upper limb [22,23]. Hardwick et al. conducted a study using large-scale meta-analysis techniques, producing quantitative and statistical results on the underlying networks of action observation, motor imagery, and action execution in neuroimaging studies. A comparison across the neural networks detected showed that motor imagery and action observation has recruited a bilateral network of premotor and parietal regions; however, this also included large parietal–occipital volumes, and more bilateral recruitment [24]. Extensive research suggested that the MNS, involved in motor imagery, is interconnected with the cerebellum by modulating the activity of cortical inhibitory interneurons with mirror properties, contributing to visuomotor matching, which underlines the mirror principle [25].

Studies on stroke patients provided evidence of neural pattern activations in the controlesional side and near the damaged brain regions [26]. In particular, the effect of mirror illusion on activity in PC was confirmed using functional near-infrared spectroscopy (fNIRS). This instrument measured the blood oxygen level dependent (BOLD) signal. The instrument analyzes the hemodynamic response based on oxygenated (HbO) and deoxygenated (HbR) blood in real time in the cerebral cortex during cognitive or visuomotor tasks [27]. It was easier to apply in a clinical setting for patients with contraindication for fMRI, including mechanical heart valves, shunt, etc. An fNIRS study confirmed the results obtained with fMRI in which ipsilateral activation of PC increased due to mirror illusion [28]. In addition, activation of the ipsilateral PC during movement was accompanied by decreased activation of the contralateral PC in healthy subjects. This issue, reported into patients, implies that the mirror illusion could balance the inter-hemispheric activation between the affected and un-affected side. It is well known that the unaffected side could inhibit the affected hemisphere due to the deterioration of motor performance. Therefore, it is assumed that MT could contribute to a synchronization of the inter-hemispheric activation balance and, through fNIRS, it was possible to observe this phenomenon during the execution of tasks. Kim et al. have also used fNIRS to investigate brain activation during MT with a robotic glove and demonstrated that robotic mirror therapy (RMT) induced a greater activation of the controlesional motor cortex in both groups of stroke and healthy subjects, creating a balanced inter-hemispheric activation during RMT [29].

In this paper, we analyzed the neural effect of MT in repetitive movements by fNIRS to measure neural activation in the brain for the MT condition and N-MT condition in post-stroke patients with upper limb hemiparesis.

## 2. Materials and Methods

### 2.1. Participants

We enrolled ten late sub-acute stroke right-handed patients who had suffered from ischemic and haemorrhagic strokes (see Table 1). All of these patients were approached in our neurorehabilitation unit at IRCCS Centro Neurolesi “Bonino Pulejo” in Messina, Italy, after few months from the stroke and providing written informed consent according to the ethical standard of the Declaration of Helsinki. The study was approved by the Local Ethics Committee of IRCCS Centro Neurolesi Bonino Pulejo. All patients followed the conventional rehabilitative treatment. The inclusion criteria were the following: (1) from 3 to 6 months from the acute event; (2) Fugl–Meyer scores for the upper limb ranging from 26 to 45 indicating moderate motor impairment; (3) Functional Independence Measure (FIM) scores ranging from 55 to 65; (4) MMSE > 20. The exclusion criteria were the following: presence of language deficits; severe visual and perception impairment; more than 6 months from the acute event.

### 2.2. Experimental Setup

**Figure 1:**
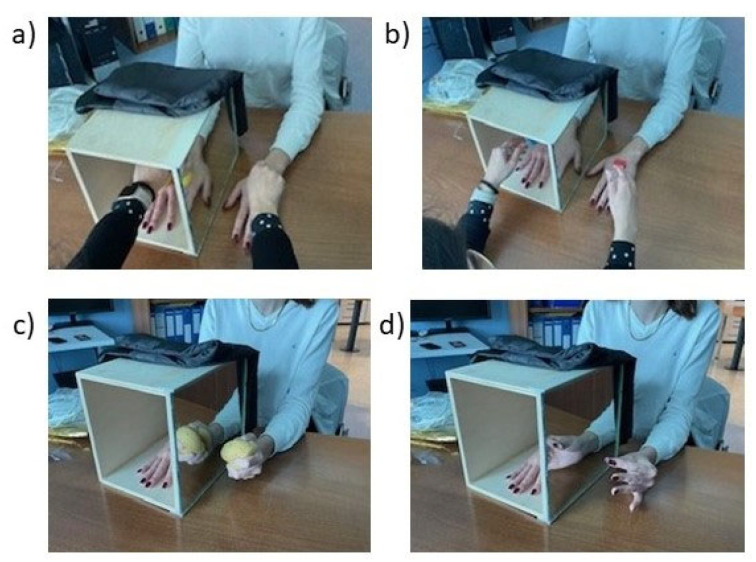
Examples of experimental tasks during MT condition: (**a**) rough brush; (**b**) smooth brush; (**c**) grasping; (**d**) finger tapping.

**Figure 2:**
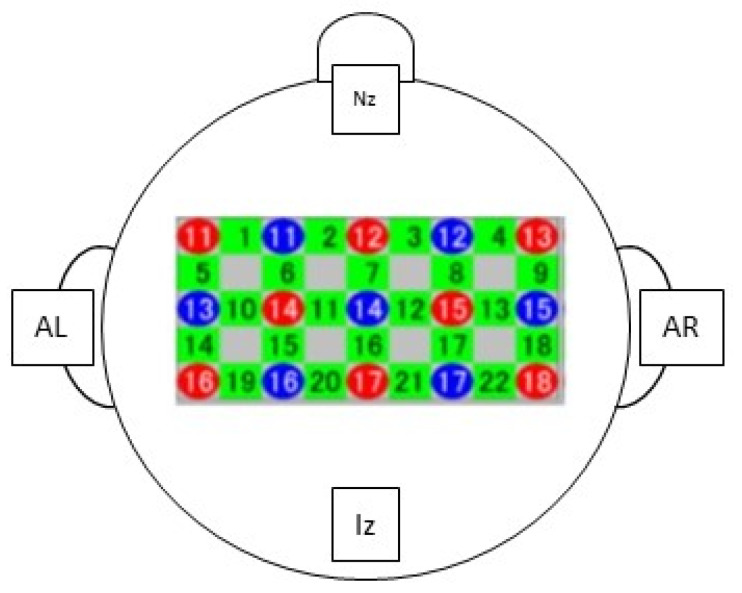
Schematic of the optode configuration matrix used and its positioning. The four reference points used are also indicated: nasion (Nz); inion (Iz); left auricular point (AL); right auricular point (RL). In the grid, the emitters are red (n. 8), the detectors are blue (n. 7); the other green point represents the acquisition channel number.

The experiment was conducted in a room located away from disturbing noise and was illuminated by artificial light. Blacked out curtains were used to avoid distractions from the outside. Experimenters assisting during the experiment included a neuropsychologist who gave instructions to the patient and conducted passive tactile stimulation and a neurophysiology technician for data acquisition with fNIRS and headcap. The instruments used for the experiment were a mirror box of wood with a height of 23.5 cm, length of 28 cm and width of 22 cm. It was opened from both sides that featured a central mirror and patients were instructed to insert the hemiplegic hand inside the box (Figure 1). The affected limb was covered by a cloth, while the unaffected limb was placed on the opposite side, so its reflection could be seen on the mirror. The MT protocol involved two motor tasks: grasping performed by the patients with a sponge and finger tapping in which patients were instructed to finger tap; the neuropsychologist showed the movement and before the recording asked the patient to try it three times (Figure 1), while the two tactile stimulation tasks performed were as follows: a rough stimulus elicited with a rough brush and smooth stimulus elicited with a silicone brush (Figure 1). Both stimuli were performed by the neuropsychologist on the skin of both the affected and unaffected upper limb simultaneously. The fNIRS was used to measure brain activation by using a Hitachi ETG-4100 NIRS system (Hitachi Medical Systems, SGP), which uses infrared light of two wavelengths (695 nm and 830 nm, respectively) to measure concentration changes in oxygenated and deoxygenated hemoglobin brain levels, with a sampling rate of 10 Hz. A configuration matrix of 3 × 5 optodes, 8 sources and 7 detectors, for a total of 22 channels, was positioned above the forehead. Based on previous findings on the role of the fronto-parietal cortices during movement mirroring [28,30], we chose optode positions to cover the prefrontal, motor and parietal cortex. A schematic of the optode positioning is shown in Figure 2. To register the positions of the optodes, which were saved in Montreal Neurological Institute (MNI) coordinates, four references were used: the nasion (Nz), the inion (Iz), the left preauricular point (AL), and the right preauricular point (AR) (see Figure 2). Data were collected using the Beer–Lambert law, whereby detectors measured the level of oxygenated haemoglobin (HbO, 850 nm) and deoxygenated hemoglobin (HBR, 760 nm) with a sampling rate of 4.4 Hz [31]. Data analysis was carried out using NIRS-SPM software (version 4, revision 1) [32,33] with the MATLAB^®^ R2009a tool (MathWorks Inc., Natick, MA, USA).

### 2.3. Experimental Procedures

**Figure 3:**
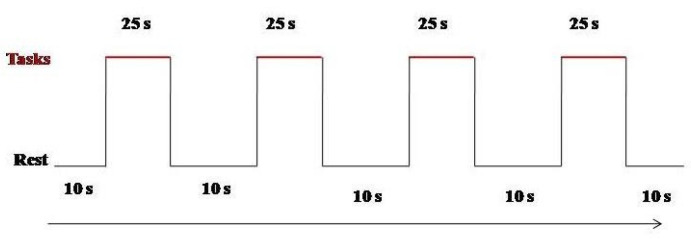
Square wave of the experimental design. The black line at the bottom represents the 10 s rest period; the red line at the top represents 25 s of active task. This design was performed for both MT and N-MT conditions for each task.

**Figure 4:**
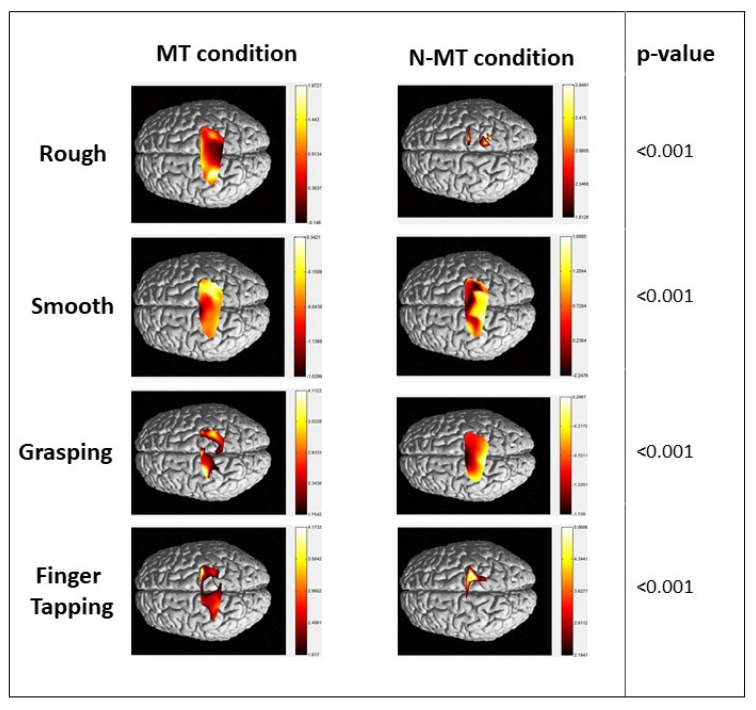
HbO concentration in right hemiparesis group. Maps of HbO during the four tasks performed.

Patients were exposed to two experimental conditions: mirror therapy condition (MT) and no mirror therapy condition (N-MT). Patients were instructed to look at the projection of their hand in the mirror in the MT condition, while seeing their hand during the N-MT condition. All participants performed motor tasks and tactile stimulation tasks at one time point in a single experimental session. The stimuli were submitted a stimulus per second. The start and end of each task were triggered by a sound. During both conditions, a mirror was placed between the two arms of the patients. The healthy upper limb was positioned on the side of the mirror, while the paretic upper limb was placed inside a box on the back of the mirror. During the performance of motor tasks, patients were asked to observe the movement of the healthy upper limb projected through the mirror in the MT condition. In the N-MT condition, the mirror was covered by a black panel, and the patients were asked to perform the same tasks without seeing their movement of the healthy upper limb in the mirror. During tactile stimulation tasks, both rough and smooth stimuli were administered by a neuropsychologist who applied the stimuli on the back of hand of both the affected and unaffected upper limb simultaneously; patients were instructed to look at their hand in the mirror and did not produce any voluntary movement. The performance was carried out in both MT and N-MT conditions. In Figure 3, we explained, in a square wave, the experimental design. It consisted of 10 s of rest, alternating for four times at 25 s of the task session (Figure 4). In particular, MT and N-MT conditions were conducted for approximately 2 min and 30 s per task, totaling 10 min for each condition. The entire experiment lasted 40 min, including the time required for the assembly of the fNIRS’s headset. The fNIRS recording was carried out once during both conditions.

### 2.4. Outcome Measurements

The measurements were conducted by a neuropsychologist for the cognitive assessment and by a physiotherapist for the motor evaluation. The evaluation was conducted at one time point before the performance of the experiment as inclusion criteria for the selection of the participants. Fugl–Meyer assessment was used to assess motor impairment, while Functional Independence Measure (FIM) for the assessment of disability. Mini Mental State Examination (MMSE) was used to select groups and evaluate the level of cognitive impairment.

- Mini Mental State Examination

The test consists of 30 items that assess orientation, memory, attention, calculation ability, language, and spatiotemporal organization. The total is 30 points and a score of 26 indicates a normal cognitive function [34].

- Fugl–Meyer Assessment

The Fugl–Meyer assessment (FMA) is a standardized instrument for evaluating hemiparesis in post-stroke patients. The motor score ranges from 0 (hemiplegia) to 100 points (normal motor performance), with 66 points for upper extremities and 34 points for the lower extremities [35].

- Functional Independence Measure

Functional Independence Measure (FIM) is used to measure different types of disability and includes measures of independence for self-care, including sphincter control, transfers, locomotion, communication, and social cognition. Each dimension is rated on a 7-point scale, with 7 indicating complete independence, 6 modified independence, 5 supervision or setup, 4 minimal contact assistance (client expends 75% or more of the effort), 3 moderate assistance (client expends between 50% and 75% of the effort), 2 maximal assistance (client expends between 25% and 50% of the effort), and 1 indicating total assistance (client expends <25% of the effort) [36].

### 2.5. Analysis

- fNIRS data pre-processing

Before any further analysis processing, data filtering was applied to extract frequency components related to the hemodynamic signal. The HbO and HbR data were filtered with the canonical HRF filter to remove high-frequency noise and temporal correlations This was carried out according to the pre-staining method, which has been shown to be more effective in calculating activation maps than the bleaching method [37,38]. Finally, baseline correction was performed again by subtracting the new value of the concentration change at the starting time.

- Measurement model

According to the Modified Beer–Lambert Law (MBLL) [39], within a consolidated model already successfully applied in the literature [40], we hypothesize that the chromophores involved are oxy- and deoxy-hemoglobin (HbO and HbR, respectively). Furthermore, the differential path-length factor (DPF) parameter d(r) can be obtained, in principle, in time-domain or frequency-domain systems by calculating the spread function of the time points [41]. Furthermore, many other conditions, such as the depth of the scalp and the shape of the head, could negatively influence the measurements obtained by the instrument, resulting in signal dispersion effects that depend on the subject.

The generalized linear model (GLM) is now a standard analysis method used for functional magnetic resonance imaging (fMRI), as well as NIRS [42,43]. It describes a quantity measured in terms of a linear combination of N explanatory variables, plus an error term. The signal can be approximated as the convolution of a stimulus function and a hemodynamic response function (HRF). For model specification, the canonical HRF composed of two gamma functions was used [37].

- Statistical Analysis

GLM incorporates the convolution of the experimental design input function with a canonical HRF to model the observed response in brain activation data. Regarding error estimation, our GLM assumes normally distributed, independent errors, and we have implemented correction procedures for multiple statistical comparisons to control the false-positive rate. This approach has allowed to generate brain maps of t-statistics, where each pixel represents a *t*-test assessing whether the activity of a given channel is significantly different from the rest phase.

A t-statistic coefficient can be calculated, as described in [38], to test the null hypothesis of no significant activation for a specific channel compared to a reference phase (generally the resting phase). In our case, we applied this analysis for both HbO and HbR, for each patient separately, using the procedure available in the NIRS-SPM software. The variables that we studied were the differences in HbO in different tasks during the MT condition and N-MT condition with an intra-group analysis. Furthermore, we performed group analysis for the two groups of patients (right hemiparesis and left hemiparesis), using the method of global alignment of the interpolated maps of the patients present in the NIRS-SPM software. The Euler expected characteristics approach was used to calculate the *p* value.

## 3. Results

- fNIRS analysis

**Figure 5:**
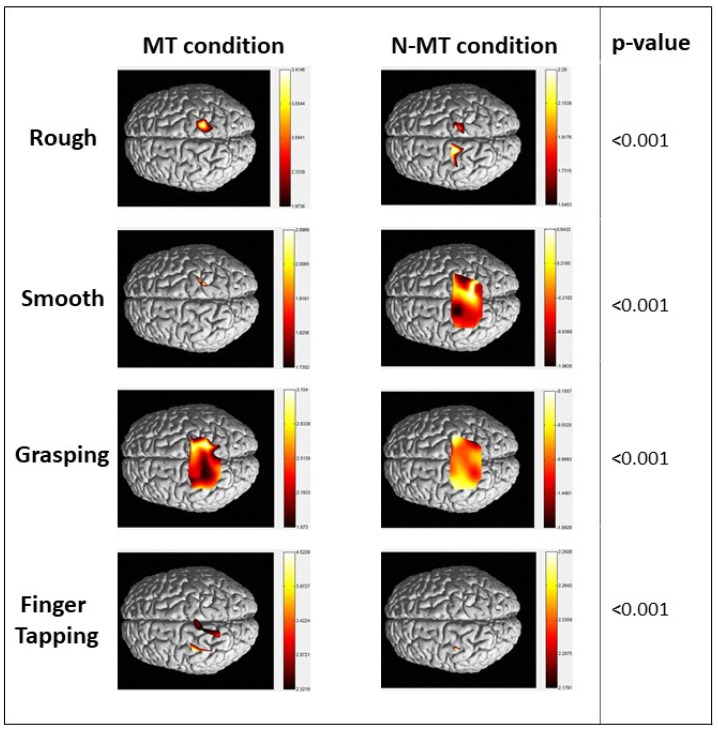
HbO concentration in left hemiparesis group. Maps of HbO during the four tasks performed.

Data collected from each patient were analyzed by GLM. These results were taken by performing an analysis of the intra-group by subdividing patients according to the side of upper limb hemiparesis. fNIRS HbO interpolated maps for each group are reported in Figure 4 for right hemiparesis and Figure 5 for left hemiparesis, respectively.

- Right hemiparesis group

The group of patients with right hemiparesis demonstrated significant HbO concentrations under both MT and N-MT conditions across all tasks (Figure 4, *p* < 0.001). Specifically, during the grasping task under MT conditions, cortical activations were observed in the central post gyrus, as well as the superior and central sulcus of both hemispheres. For the finger tapping task in the MT condition, significant activations were highlighted in the precentral sulcus, postcentral sulcus, and superior gyrus of both hemispheres (Table 2). Notably, during tactile stimulation, there were significant activations in the precentral gyrus, and the central, postcentral, and superior sulcus, according to the Montreal Neurological Institute (MNI) coordinates (Table 2).

- Left hemiparesis group

In the MT condition, the left hemiparesis group displayed lower and more lateralized HbO concentrations during rough, smooth, and finger tapping tasks. This group exhibited significant differences in HbO concentrations between MT and N-MT conditions across all tasks (Figure 5, *p* < 0.001). Notably, patients with left hemiparesis showed brain activations in both hemispheres, including the central sulcus, precentral gyrus, and postcentral gyrus during the grasping task. The finger tapping task revealed more localized, less extensive, and less intense activation in the left central and postcentral gyrus in the MT condition. In contrast, tactile stimulation in the MT condition prompted more focused activations in the central sulcus and the right precentral gyrus (Table 3), according to Montreal Neurological Institute (MNI) coordinates.

## 4. Discussion

In this study, we aimed to analyze the neural effect of MT in motor and tactile stimulation tasks by fNIRS to measure neural activation in the brain. The results showed central, pre-central and post-central area activations corresponding to activations in fronto-parietal brain regions as well as sensorimotor areas. By reflecting the movement of the heathy hand, motor areas involved in the acute event were activated, deceiving the brain. This type of hand rehabilitation system allowed users to train damaged brain circuits and recruit new neuronal units for hemiplegic limb recovery through the healthy hand. Our findings showed that MT increased neural activity in both the contralateral and ipsilateral sensorimotor cortex. Although many studies have demonstrated that robotic therapy induces better functional recovery compared to conventional therapy [44,45], MT involves patients’ intention in using their residual motor skills to promote functional recovery and a larger neural involvement of the proximal and contralateral motor cortex of the affected hand. A study conducted in healthy right-handed subjects demonstrated that MT was more effective if participants used their dominant hand during a grasping task. Specifically, they showed a significant decrease in the laterality index compared to using the left hand, where the lateralization of cerebral activation did not shift [46]. We need to more thoroughly explore the significance of the differences observed between MT and N-MT. Specifically, our results suggested that not only the presence of the mirror or not but also the type of motor activity (e.g., grasping vs. finger tapping) could play a role in rehabilitative outcomes. For instance, the cerebellum could be especially relevant in activities such as finger tapping, where fine motor control and coordination are key [25]. This was supported by our results because many of the areas that are activated are critically connected to the cerebellum, which plays a fundamental role in the MNS. This assumption could be crucial to explain the cerebral activation in the conditions of tactile stimuli to the back of the hand. It is well known that the brain’s lateralization mechanism is specific to the dominant hand, probably due to the varying manipulability of the dominant and non-dominant hands. For this reason, stroke patients could experience motor illusions more easily in the MT condition than healthy subjects, which our study supports. Furthermore, hemispheric lateralization appears to be a crucial factor in understanding these differences between our patients’ groups. Literature studies suggest that the left hemisphere, which is often dominant for visuo-motor control, may contribute differently to the recovery process compared to the right hemisphere, particularly in tasks requiring precise motor planning and execution [47]. Our findings indicated that these hemispheric differences could partially explain the variation in the effectiveness of MT versus N-MT for different activities, underscoring the importance of considering both the type of therapy and the nature of the motor task in designing rehabilitation programs. In addition, despite brain damage, neuronal activation involved both the healthy hemisphere and the brain regions surrounding the injured hemisphere, demonstrating that MT could be functional for neural reorganization. In the right hemiparesis group, the MT condition showed greater activation during the “grasping” and “finger tapping” tasks (Figure 4, Table 2). The illusion of the mirror reflection helps the system to rearrange and modulate the motor circuits normally involved by recruiting not only the areas in proximity to the lesion but also the contralateral side (see Figure 4). In the group of patients with left hemiparesis, the “grasping” task (see Figure 5, Table 3) seemed to produce better results in terms of intensity of activation and areas involved, compared to other tasks. This could be due to the fact that the lesion affected the motor areas of the non-dominant hemisphere, as previously explained. However, the tasks mainly involve motor and sensorimotor components due to the involvement not only of the movement, but also of the visual feedback given by the mirror. Recent evidence has shown that EEG correlates of action observation, particularly event-related desynchronization (ERD), are an early predictor of motor recovery in subcortical stroke. This finding is particularly relevant to our study on MT that involves the observation of movements that activate motor-related brain areas [48]. In the context of MT, patients observing the reflection of their intact limb’s movements may similarly experience ERD, which could serve as a neural marker of motor recovery. The ERD during action observation and the neural processes of MT suggest that early EEG changes, such as ERD, could potentially be used to predict which patients are more likely to benefit from MT. This supports the hypothesis that the engagement of the motor system through observation, whether in traditional action observation or through MT, plays a critical role in post-stroke rehabilitation.

In this light, our results demonstrate that MT not only facilitates motor recovery but also engages early motor cortex activation, as evidenced by studies on ERD. According to previous findings, early motor cortex engagement, detected through EEG during action observation, is predictive of functional recovery [49]. Thus, further studies could investigate whether EEG-based monitoring of ERD during mirror therapy could serve as a biomarker for predicting rehabilitation outcomes. Therefore, the fNIRS tool could provide other neuroanatomical information related to EEG findings to analyze prognostic and therapeutic aspects of therapy. Promising results were found with kinematics and neurophysiological data in stroke patients. An EEG study showed that during these measures, the healthy hemisphere tends to favor the affected side. These results suggested the importance of considering kinematics measures as kinematics biomarkers to evaluate motor recovery in stroke patients [50]. The identification of biomarkers, neurophysiological and kinematics in the early stage of stroke is crucial in order to choose the best rehabilitation treatment and maximize the improvement of synaptic plasticity especially in the first few months after the acute event.

It is well known that neural plasticity is responsible for functional recovery [51,52]. Cortical plasticity has been demonstrated in terms of reorganization, remodulation of brain activity and expansion of topographic maps through motor, sensorimotor rehabilitative treatments [53] and motor imagery [54,55,56].

However, the exact mechanism behind this phenomenon remains unclear. MT has been shown to play an interesting role in inducing neural plasticity. Previous studies have demonstrated the involvement of motor areas and sensorimotor cortex in motor recovery after a stroke. Therefore, it would be interesting to consider the effects of MT on motor area activation and how it contributes to the recovery. However, this study presented few limitations. First, the small number of participants makes it difficult to generalize the results. Second, there is no follow-up assessment in this study. Third, all patients in this study were right-handed, with around 70% of subjects having a right affected side and 30% having a left affected side. Finally, not all patients were screened with an MRI due to the presence of mechanical valves or shunts. For these reasons, it was not possible to use the lesion overlap map.

## 5. Conclusions

This research has the potential to improve the assessment of new guidelines for developing rehabilitative projects to help post-stroke patients increase neuronal stimulus, leading to better motor recovery. To ensure the accuracy of future research, the next step could be to implement rehabilitative training during patients’ hospitalization, based on the principles of MT, and evaluate its effectiveness as a rehabilitation tool.

In this pilot study, we highlighted the neural mechanisms that underlie the improvement of upper limb function using the MT technique. The recorded neurophysiological response contributed to our understanding of the mechanisms behind a new motor representation of movement in stroke patients. In particular, fNIRS has proven to be a useful tool for monitoring the re-modelling of neuronal networks and changes in cortical activity during the MT condition. Few studies have been conducted with the fNIRS system and MT. The increase in the number of samples could enable us to consider more stable neural patterns of response to this therapy in stroke patients to predict their recovery. In addition, previous studies did not distinguish between the side of paresis and hemisphere affected. In fact, in future studies, our aim is to distinguish patients based on the affected side to investigate the differences in MT responses considering the hemispheric specialization.

## Figures and Tables

**Table 1:** Clinical characteristics of stroke subjects.

Patients	(n = 10)
Age (years)	61.25 ± 6.89 ^a^
Sex (male/female)	7/3
Disease Duration (month)	3 ± 1.22 ^a^
Etiology (ischemia/haemorrhage)	4/6
Affected upper limb (left/right)	7/3
MMSE (score)	20.27 ± 4.46 ^a^
Fugl-Meyer (score)	39 ± 6.32 ^a^
FIM (score)	55 ± 7.84 ^a^
Stroke location (right hemisphere)	Fronto-parietal haemorrhage (n = 3) Capsular nucleus ischemia (n = 2) Fronto-temporal hemorrhage (n = 2)
Stroke location (left hemisphere)	Capsular nucleus ischemia (n = 2) Fronto-temporal haemorrhage (n = 1)

^a^ Mean ± standard deviation (SD); MMSE = Mini Mental State Examination; FIM = Functional Independence Measure.

**Table 2:** MT condition in stroke patients with right hemiparesis.

Tasks	Cerebral Activation Areas	Threshold T	*p*-Value
Rough	Precentral gyrus, central sulcus, superior post-central gyrus right and left side	0.83	<0.001
Smooth	Precentral gyrus, central sulcus, superior post-central gyrus right and left side	0.15	<0.001
Grasping	Post-central gyrus, central sulcus right and left side	2.93	<0.001
Finger tapping	Precentral gyrus, post-central gyrus left side, superior post-central gyrus right side	2.89	<0.001

**Table 3:** MT condition in stroke patients with left hemiparesis.

Tasks	Cerebral Activation Areas	Threshold T	*p*-Value
Rough	Right central sulcus	3.05	<0.001
Smooth	Right pre-central gyrus	2.20	<0.001
Grasping	Precentral gyrus, superior post-central gyrus right and left side	2.30	<0.001
Finger tapping	Left central and post-central gyrus	2.42	<0.001

## Data Availability

The datasets used and/or analyzed during the current study available from the corresponding author on reasonable request.
